# Importance of surface morphology on secondary electron emission: a case study of Cu covered with carbon, carbon pairs, or graphitic-like layers

**DOI:** 10.1038/s41598-023-34721-8

**Published:** 2023-05-22

**Authors:** L. Diaz, A. Karkash, S. Alsharari, R. P. Joshi, E. Schamiloglu, M. Sanati

**Affiliations:** 1 Department of Physics and Astronomy, Texas Tech University, Lubbock, TX 79409 USA; 2 Department of Electrical and Computer Engineering, Texas Tech University, Lubbock, TX 79409 USA; 3 Department of Electrical and Computer Engineering, University of New Mexico, Albuquerque, NM 87131 USA

**Keywords:** Engineering, Physics

## Abstract

Understanding the relationship between surface adsorbates and secondary electronic emission is critical for a variety of technologies, since the secondary electrons can have deleterious effects on the operation of devices. The mitigation of such phenomena is desirable. Here, using the collective efforts of first-principles, molecular dynamics, and Monte Carlo simulations, we studied the effects of a variety of carbon adsorbates on the secondary electron emission of Cu (110). It was demonstrated that the adsorption of atomic C and C$_2$ pair layers can both reduce and increase the number of secondary electrons depending on the adsorbate coverage. It was shown that under electron irradiation, the C–Cu bonds can be dissociated and reformed into C$_2$ pairs and graphitic-like layers, in agreement with experimental observation. It was verified that the lowest secondary electron emission was due to the formation of the graphitic-like layer. To understand the physical reason for changes in number of secondary electrons for different systems from an electronic structure perspective, two-dimensional potential energy surfaces and charge density contour plots were calculated and analyzed. It was shown that the changes are strongly influenced by the Cu surface morphology and depends highly on the nature of the interactions between the surface Cu and C atoms.

## Introduction

Electronic emission is the ejection of electrons from a material in response to some physical stimuli such as heat or an external electric field. Secondary electron emission (SEE) occurs when a material is bombarded with sufficiently energetic charged particles^1–8^ resulting in the ejection of secondary electrons. The generation of secondary electrons can have deleterious effects on electronic devices and output of experiments performed in vacuum such as those involving rf/microwave generators and particle accelerators^9^. The collisions of primary electrons with accelerator walls can result in the emission of secondary electrons that can subsequently impact surfaces again and continue charge generation. This multiplication of electrons can resonantly interfere with the temporal and spatial structure, thereby, having a destructive effect on beam quality. This phenomenon is known as electron cloud buildup^9^. Therefore, alleviating the generation of secondary electrons in such systems is critical to maintaining functionality.

Despite best efforts, all material surfaces have contaminants with the two most prevalent being carbon and oxygen^10^. Indeed, previously it was shown that the formation of CO and CO$_2$ layers on the surface significantly enhanced the secondary electron yield (SEY) of the system^10–13^. Therefore, to reduce secondary electron generation (i.e., reduce the SEY) one needs to clean and remove CO, CO$_2$, and any other detrimental contaminants that may be present from the surface. This is frequently achieved by baking the sample at high temperatures (typically around 100–300 °C)^10^ and/or irradiating the surface to dissociate the adsorbed contaminants^9,14,15^. However, during the irradiation process, the removal of the contaminants can also be accompanied by a graphitization process due to residual and dissociated C atoms. This process has been observed experimentally^9,10,16,17^. In general, it is known that the formation of C layers (graphitization) reduces the SEY of the material surface^17–19^. Additionally, by increasing the irradiation dose, a further decrease of the secondary electrons was achieved where the least reported value of the SEY peak corresponded to impinging electrons with a kinetic energy of 500 eV^9^. In spite of the available experiments on C/Cu systems^17^, the physical reasons behind the SEY reduction or relation between the SEY and the different forms of C adsorption (single atomic layer, C$_2$ pair formation, or graphite layers etc.) are still unknown. It was suggested^9^ that a systematic study was needed to fully understand the effect of surface conditioning on the generation of secondary electrons and to uncover the inherent and underlying physics.

Using a blend of first-principles calculations, Monte Carlo (MC) methods, and molecular dynamics (MD) simulations, the SEY of the adsorption of C (atomic, C$_2$ pairs, and graphitic-like) on the Cu (110) surface was systematically studied. In this study, Cu was chosen as the substrate material because of its utility in high power applications and abundant availability of experimental data. First-principles calculations were used to identify the most stable structures and material parameters required for MC and MD simulations. Previously, it was shown that formation of graphitic-like layers can reduce the SEY of Cu^9^; however, to our knowledge no studies for C atomic or C$_2$ pair layers are available. In agreement with experimental observation^9^, MD simulations performed in this work confirmed that electron irradiation can cause the dissociation of C atoms from the Cu surface and lead to the formation C$_2$ pairs. Interestingly, increasing C$_2$ pair coverage reduced the SEY, while increasing atomic C coverage exhibited the opposite trend. Furthermore, continued electron irradiation of the C$_2$/Cu system resulted in the formation of a graphitic-like adsorbate layer^9,17^. To study the effects of a graphitic-like structure, an energetically stable six monolayer (ML) C configuration was generated and shown to reduce the SEY further in the present simulation work. Using a combination of two-dimensional potential energy surfaces and charge density contour plots, here we show how the surface morphology of the Cu surface is directly responsible for the changes in SEY for all systems.

## Results

To simulate the secondary electron emission (SEE), a Cu (110) surface was created instead of the polycrystalline structures typically used in experiment. This replacement is justified since the the low-index surface with the lowest work function most closely resembles the work function and surface properties of the polycrystalline surface^20,21^. The calculated and experimental work functions for clean Cu (110) were in good agreement with $\varphi _{cal}=4.40$ eV (4.48^22^). To investigate the effects of the C adsorption entirely, numerous atomic configurations were examined for atomic C, C$_2$ pair, and graphitic-like layers. The stability of each system was confirmed by calculating the adsorption energy of the system where a negative energy corresponds to a stable adsorbate-substrate interaction. The adsorption energy ($E_{ads}$) was obtained from

1 $$\begin{aligned} E_{ads} = \frac{1}{N}(E_{sys} - E_{slab}) - \frac{1}{2}E_{C_2}, \end{aligned}$$

where $E_{sys}$, $E_{slab}$, and $E_{C_2}$, are the total energies of the C/Cu (110) system, clean Cu (110) slab, and the C$_2$ molecule, respectively. N is the number of C atoms per unit cell. For the C$_2$ molecule, the calculated bond length and binding energy were 1.31 Å and − 7.01 eV, respectively, which are comparable with the measured values of 1.255 Å^23^ and − 6.296 eV^24^ (bond dissociation energy).

**Figure 1:**
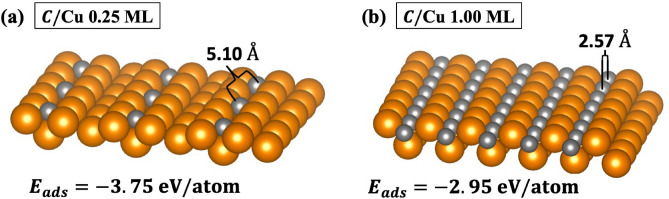
Space-filling representations of the C/Cu (110) surface with coverages (**a**) 0.25 and (**b**) 1.00 ML. The calculated work functions for C/Cu at 0.25 and 1.00 ML coverages were 4.90 eV and 4.89 eV, respectively. Gold and gray spheres represent the Cu and C atoms, respectively. These structures were made using VESTA^25^.

### Adsorption of a carbon monolayer

After studying the different sites for the (110) surface, it was determined that the C atoms occupied the hollow adsorption site for the 0.25 monolayer (ML) coverage (Fig. 1a). A small surface reconstruction occurred that shifted the C adsorption site from the hollow to the long-bridge site (Fig. 1b) when increasing the atomic C coverage to 1.00 ML due to the creation of internal strain. The shift did not change the number of C nearest neighbors (four Cu atoms). However, displacement of surface atoms caused the C-Cu distance to increase by roughly 16.9% with respect to the 0.25 ML system. This increasing bond length could reduce the hybridization between Cu *d* and C *sp* orbitals resulting in weaker Cu–C bonds for the 1.00 ML system. This was verified by comparing the calculated adsorption energies of these systems for C/Cu 0.25 ($E_{ads}=-3.75$ eV/atom) and 1.00 ($E_{ads}=-2.95$ eV/atom) ML coverages. The lower adsorption energy (more negative) calculated for the 0.25 ML coverage system indicates a more stable bond between the C and Cu atoms with respect to the 1.00 ML coverage.

The MC simulation describes the incident (primary) electrons as they traverse through the host material. Along their path, primary electrons experience repetitive elastic and inelastic collisions, thereby undergoing energy loss after each collision. The energy loss and collisions are quantified by the stopping power and energy dependent inelastic mean free path, respectively. As a result of this energy exchange, secondary electrons are produced and traverse along their own path while also deflecting and scattering within the material. In the event that a secondary electron migrates to the material surface, the electron can be emitted if it has sufficient energy to overcome the surface potential barrier (work function).

The inelastic mean free path (IMFP) is critical to capturing the correct physics for the SEY calculations and was determined using the extended Mermin method. The IMFP approach used here employs the electron energy loss function (ELF)^26^, where the ELF was calculated using the dielectric tensor [$\varepsilon (q,\omega )$] obtained from density functional theory (DFT) methods^27^. The inverse-IMFP was obtained using the wavevector dependent dielectric tensor [$\varepsilon (q, \omega )$], also known as the wavevector dependent harmonic correction, using

2 $$\begin{aligned} \lambda ^{-1}_{harm} = \frac{1}{\pi a_0 E} \int _{0}^{\infty } d(\hbar \omega ) Im \bigg [ \frac{-1}{\varepsilon (q, \omega )} \bigg ] ln\bigg [\frac{2\sqrt{E-E_F-\hbar \omega }}{\sqrt{E}-\sqrt{E-2\hbar \omega }}\bigg ], \end{aligned}$$

where $\hbar$, $a_0=\frac{4\pi \varepsilon _0 \hbar ^2}{m_e e^2}=5.29\times 10^{-11}$ m, and *E* are the Planck constant, Bohr radius, and electron energy, respectively. Alternatively, the inverse-IMFP can be simplified by employing a wavevector independent form of the dielectric function [i.e., $\varepsilon (q, \omega ) \equiv \varepsilon (\omega )$]. This non-harmonic wavevector independent inverse-IMFP ($\lambda ^{-1}$) is expressed as

3 $$\begin{aligned} \lambda ^{-1} = \frac{\hbar }{\pi a_0 E} \int _{0}^{\infty } d(\omega ) Im \bigg [ \frac{-1}{\varepsilon (\omega )} \bigg ] ln\bigg (\frac{\sqrt{E}+\sqrt{E-\hbar \omega }}{\sqrt{E}-\sqrt{E-\hbar \omega }}\bigg ). \end{aligned}$$

**Figure 2:**
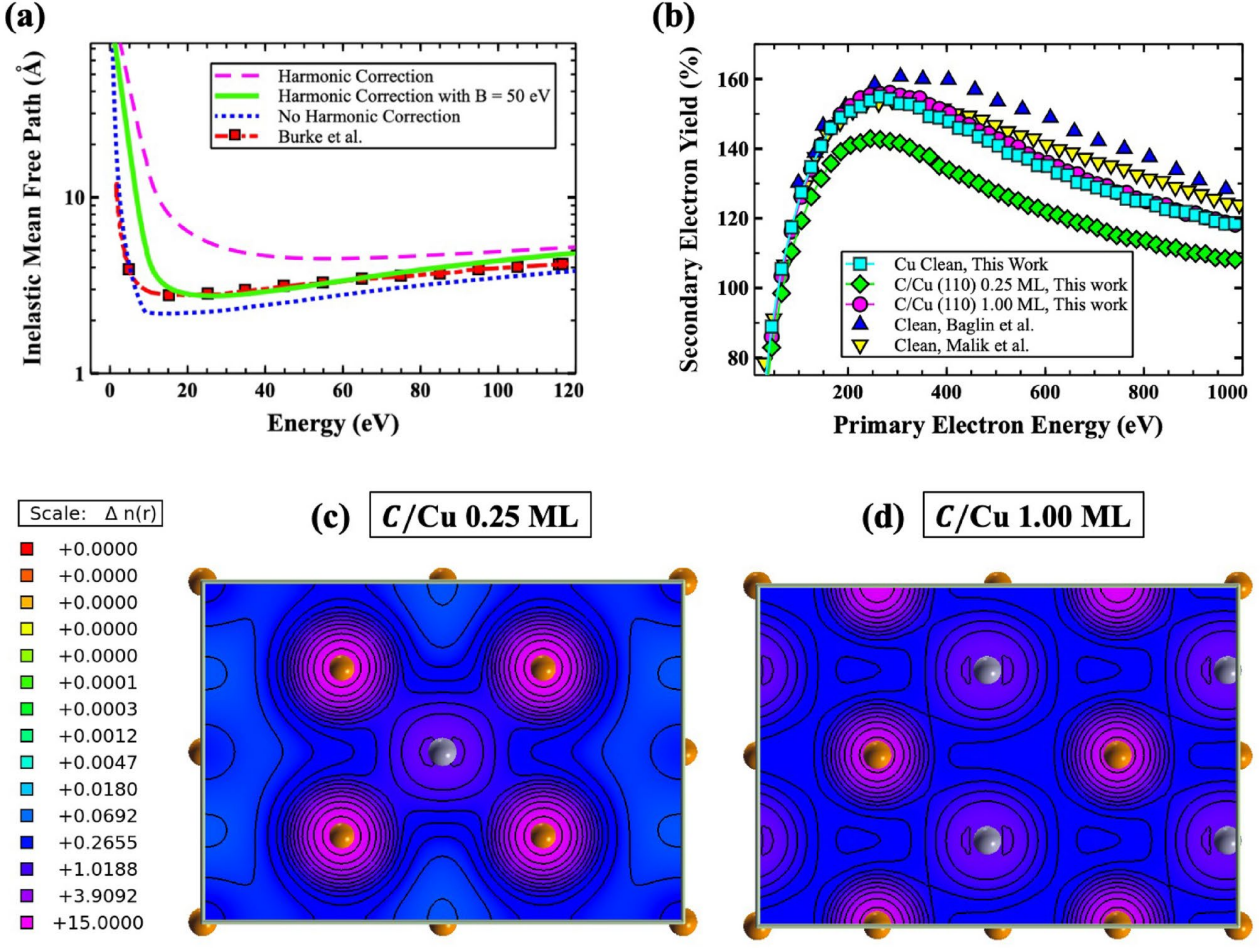
(**a**) Calculated inelastic mean free path (IMFP) for clean Cu (110) with and without the harmonic correction. Additionally, the corrected IMFP ($\lambda _e$), calculated using the harmonic correction with $B=50$ eV, is shown along with an experimental measurement for comparison^28^. (**b**) Secondary electron yield for clean Cu and C/Cu at 0.25 and 1.00 ML coverages. Experimental results are also provided for clean Cu from Baglin et al.^30^ and Malik et al.^12^. Calculated charge density [n(r) (e/Å$^3$)] contour plots for the outermost C/Cu (110) layer at (**c**) 0.25 and (**d**) 1.00 ML coverages.

To investigate the effects of the harmonic correction, the IMFP was calculated with and without the wavevector dependence and compared with the experimental measurements for Cu^28^ (Fig. 2a). As one can see from the figure, the non-harmonic and harmonic IMFPs underestimate and overestimate the experimental measurements, respectively. This overestimation of the harmonic IMFP (Mermin approach) has been observed in previous works^29^. To remedy this effect, an extended Mermin method based on the work by Da et al.^26^ was implemented where the corrected IMFP ($\lambda _e$) takes the form

4 $$\begin{aligned} \lambda _e = \lambda _{harm}[1-e^{-E/B}], \end{aligned}$$

where *E* is the electron kinetic energy. The *B* is a parameter dependent on the material of the study. In Fig. 2a, the IMFP with the harmonic correction and $B=50$ eV is compared with the previously discussed IMFPs and experimental measurements. Due to the excellent agreement with experimental measurements^28^, the harmonic correction with a $B=50$ eV was implemented for all systems in this work. In addition to the IMFP, the stopping power (energy loss per unit length experienced by an electron along their path length R) is critical to determining the SEY within the MC simulation. The stopping power (*dE*/*dR*) is given by

5 $$\begin{aligned} \frac{dE}{dR} = \frac{\hbar }{\pi a_0 E} \int _{0}^{\frac{E}{\hbar }} (\hbar \omega ) d(\hbar \omega ) Im \bigg ( \frac{-1}{\varepsilon (\omega )} \bigg ) ln\bigg (\frac{\sqrt{E}+\sqrt{E-\hbar \omega }}{\sqrt{E}-\sqrt{E-\hbar \omega }}\bigg ). \end{aligned}$$

It should be mentioned that the harmonic correction was not utilized when calculating the stopping power. Despite the improved agreement with the experimental IMFP, the stopping power did not exhibit such an improvement; therefore, no harmonic correction was used.

The SEY modeling results for the clean Cu, 0.25 ML C/Cu, and 1.00 C/Cu systems are provided in Fig. 2b along with available experimental measurements for comparison. Interestingly, one can see from Fig. 2b, the C coverage reduced the SEY significantly in going up to 0.25 ML, while increasing the coverage from 0.25 to 1.00 ML increased the SEY by roughly 14% with respect to the clean Cu surface. The physical reason for these changes is directly related to the nature of the bonding between the C and Cu surface atoms. The strong bonding between the C and Cu surface atoms reduced the probability of escaping electrons from the surface resulting in a decreased number of secondary electrons. Therefore, as it was discussed previously and confirmed by the contour charge density analysis of the 0.25 (Fig. 2c) and 1.00 (Fig. 2d) ML coverages, the surface reconstruction, caused by increasing the C coverage, weakened the C–Cu bonds resulting in an overall increase of the SEY (Fig. 2b).

### Carbon monolayer under electron irradiation

**Figure 3:**
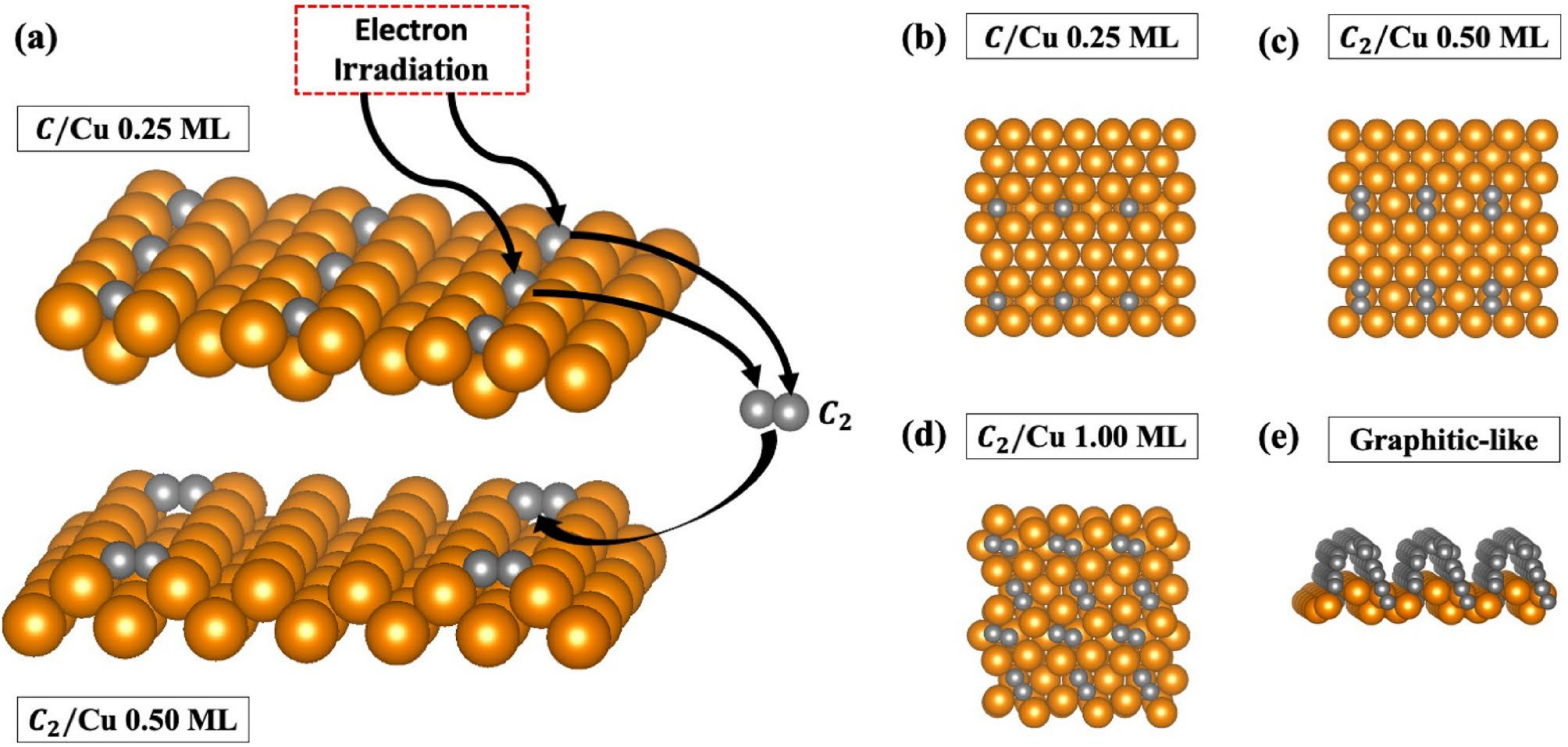
(**a**) Dissociation of C–Cu bonds (C/Cu) and the formation of C$_2$ pairs (C$_2$/Cu) on the Cu (110) surface caused by the electron irradiation. Additionally, structures for (**b**) C/Cu at 0.25 ML coverage, C$_2$/Cu at (**c**) 0.50 and (**d**) 1.00 ML coverages, and (**e**) the graphitic-like adsorbate structure are shown. Gold and gray spheres represent the Cu and C atoms, respectively. These structures were made using VESTA^25^.

For SEY measurements of a clean surface, the removal of contaminants such as O and C is necessary to obtain accurate results. In some instances, as shown in Fig. 3a, electron irradiation is used to remove O and C from Cu surfaces by dissociating the Cu–O and Cu–C bonds; however, this process is typically accompanied by the formation of C–C (*sp*$^2$) bonds^9^. Interestingly, the created C–C bonds are stronger than the existing Cu–C bonds, indicating that the electron irradiation can result in a more stable system (lower adsorption energy) than the atomic C monolayer systems. Furthermore, it was reported that high-energy electron irradiation can also result in the formation of thin graphitic-like layers^9,17^.

To study the formation of the C–C bonds and their effect on the SEY of the system, a C$_2$ pair molecule, at 0.50 and 1.00 ML coverages, and a six layer C structure (graphitic-like structure) were adsorbed onto a Cu surface. Using first-principles calculations, the most stable atomic configurations for each system was obtained. For the C$_2$ pair, it was determined that the most energetically stable adsorption site for both coverages was, analogous to the C/Cu system, the hollow site (Fig. 3b–d). The graphitic-like structure resulted in a surface reconstruction and therefore did not have a well-defined adsorption site (Fig. 3e).

**Table 1:** Calculated adsorption energies $E_{ads}$ (eV/atom), $C{-}C$ distance (Å), and calculated work functions $\varphi$ (eV) for clean Cu, C$_2$/Cu at 0.50 and 1.00 ML coverages, and for the graphitic-like structure.

Material	$E_{ads}$ (eV/atom)	$C{-}C$ Distance (Å)	$\varphi _{cal}$ (eV)
*Cu*	n/a	n/a	4.40 (4.48^22^)
$C_2/Cu$ (0.50 ML)	− 4.74	1.30	4.75
$C_2/Cu$ (1.00 ML)	− 4.50	1.32	4.91
Graphite	− 6.56	1.37–1.52	7.63

**Figure 4:**
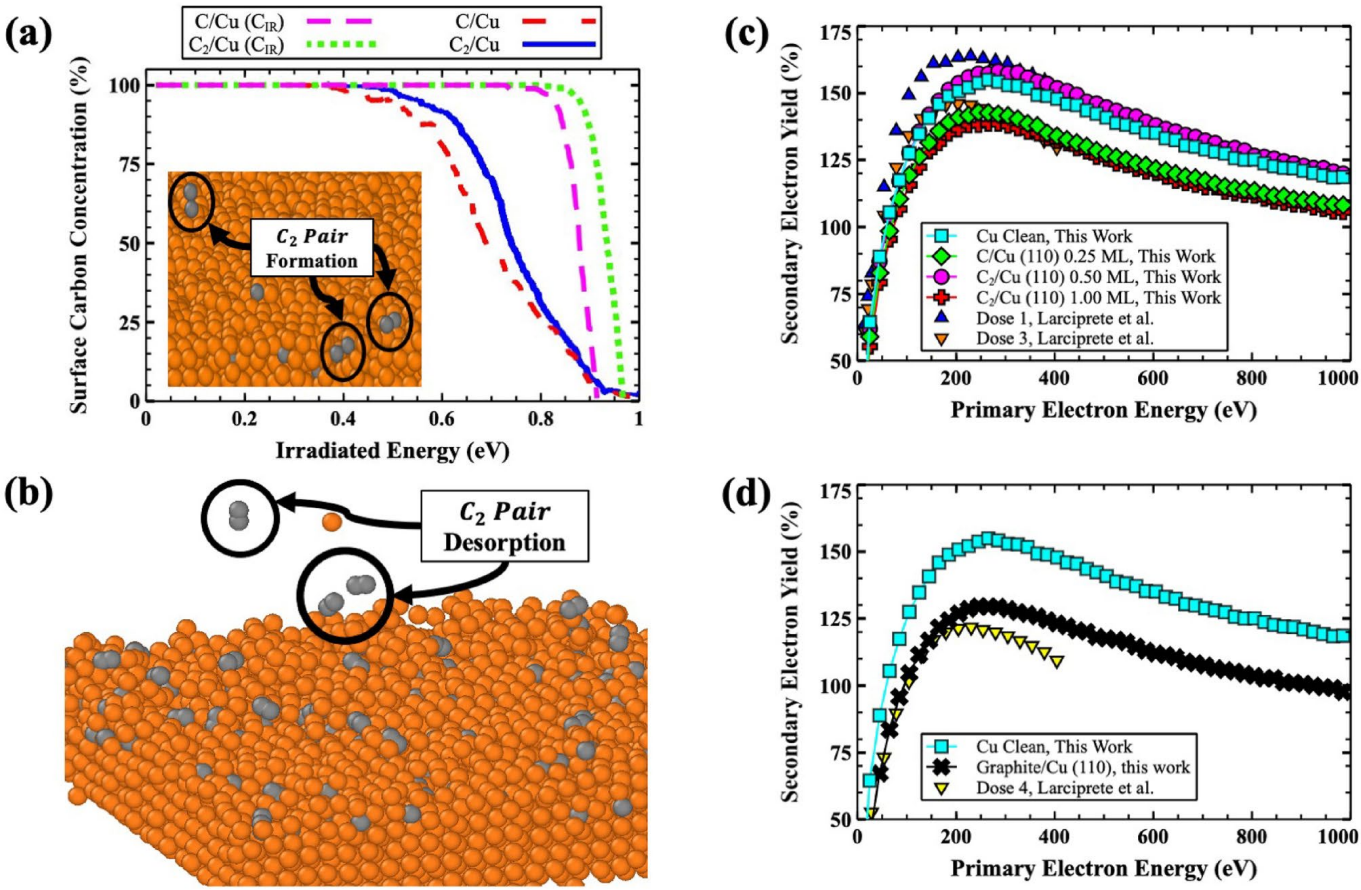
(**a**) MD simulation of the adsorbate concentration dissociated from the Cu surface as a function of irradiated energy. Two schemes are considered which included increasing the energy of carbon only (C$_{IR}$) and both C and Cu (outermost and first sublayer). The inset shows a snapshot of the C$_2$ pair formation from C/Cu surface irradiation. (**b**) Visualization of the C$_2$ pair formation and desorption at C/Cu surface at higher energies. The gold and gray spheres are representative of the Cu and C atoms, respectively. Both structures was created using OVITO^31^. (**c**) Calculated secondary electron yield for clean Cu, C/Cu at 0.25 ML coverage, $C_2$/Cu at 0.25 and 1.00 ML coverages, and (**d**) clean Cu and the graphitic-like adsorbate system are shown. All experimental measurements are from Reference^9^.

The stability of each system (C/Cu 0.25 ML and C$_2$/Cu 0.50 ML) and formation of C$_2$ pairs from the C atoms, due to electron irradiation, were studied using two different MD simulations performed based on the surface structures provided from first-principles calculations (Fig. 3b,c). In Fig. 4a, the carbon concentration is shown for two different irradiation schemes. In the first case only C atoms are irradiated while the Cu atoms kept at 300 K temperature. In the second case, the C atoms, the topmost, and first Cu sublayer are irradiated while the remaining Cu atoms temperature fixed at 300 K. The results of MD simulations reveal that at higher energies the C$_2$/Cu system has a higher C concentration indicating that the C$_2$/Cu system is more stable than the C/Cu system. These predictions are in agreement with the calculated adsorption energies $E_{ads}=-3.75$ and $E_{ads}=-4.74$ eV/atom for C/Cu 0.25 ML and C$_2$/Cu 0.50 ML (Table 1), respectively. Additionally, closer examination of the C/Cu simulation confirmed the formation of the C$_2$ pair, from initially isolated atomic C atoms, onto the surface was observed as shown in the inset of Fig. 4a,b. Therefore, the C atoms can dissociate from the Cu surface, form C$_2$ pairs, and create the C$_2$/Cu system. At higher energies, the C$_2$ concentration for both C/Cu and C$_2$/Cu systems have become similar to each other (Fig. 4a). It is important to mention that the surface desorption of C atoms are in the form of C$_2$ pairs (Fig. 4b). For this reason, the surface adsorption energies are measured with respect to C$_2$ binding energy (Eq. 1).

**Figure 5:**
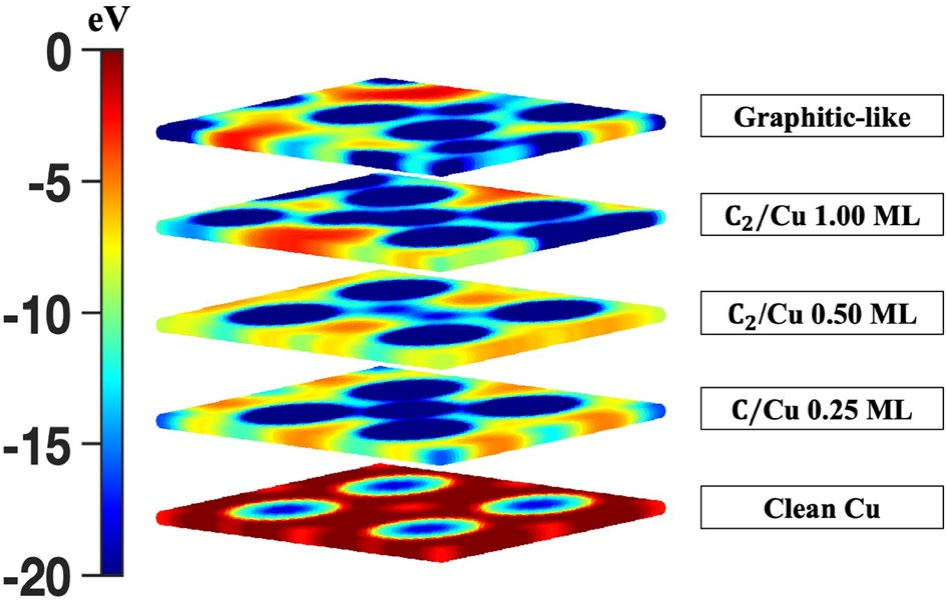
Calculated two-dimensional potential energy surfaces for the outermost layer of the clean Cu, C/Cu at 0.25 ML coverage, C$_2$/Cu at 0.50 and 1.00 ML coverages, and the graphitic-like adsorbate system are shown. Additionally, a potential energy thermostat is provided to compare areas of low and high potential energy.

In Fig. 4c,d, the calculated SEY are shown for the C/Cu (0.25 ML), C$_2$/Cu (0.50 and 1.00 ML), and graphitic-like/Cu systems compared with available experimental measurements^9^. For all systems, the calculated SEY values are in excellent agreement with the experimental observations from Larciprete et al. who treated their systems with doses of irradiation (doses 1 and 3)^9^. Interestingly, contrary to the C/Cu case, by increasing the C$_2$ coverage from 0.50 (Fig. 3c) to 1.00 (Fig. 3d) ML the maximum SEY decreased by close to 19.5% (Fig. 4c). Additionally, the formation of the 0.50 and 1.00 C$_2$ monolayers and graphitic-like layers on the Cu surface resulted in a maximum SEY change of nearly 3%, − 16.5%, and − 26% (Fig. 4d), respectively, compared to the clean Cu surface. Therefore, as confirmed by experimental observation^9^ and verified here, subsequent irradiation of the system (increased dosage) will result in the formation of more C–C bonds; thereby, reducing the SEY further.

**Figure 6:**
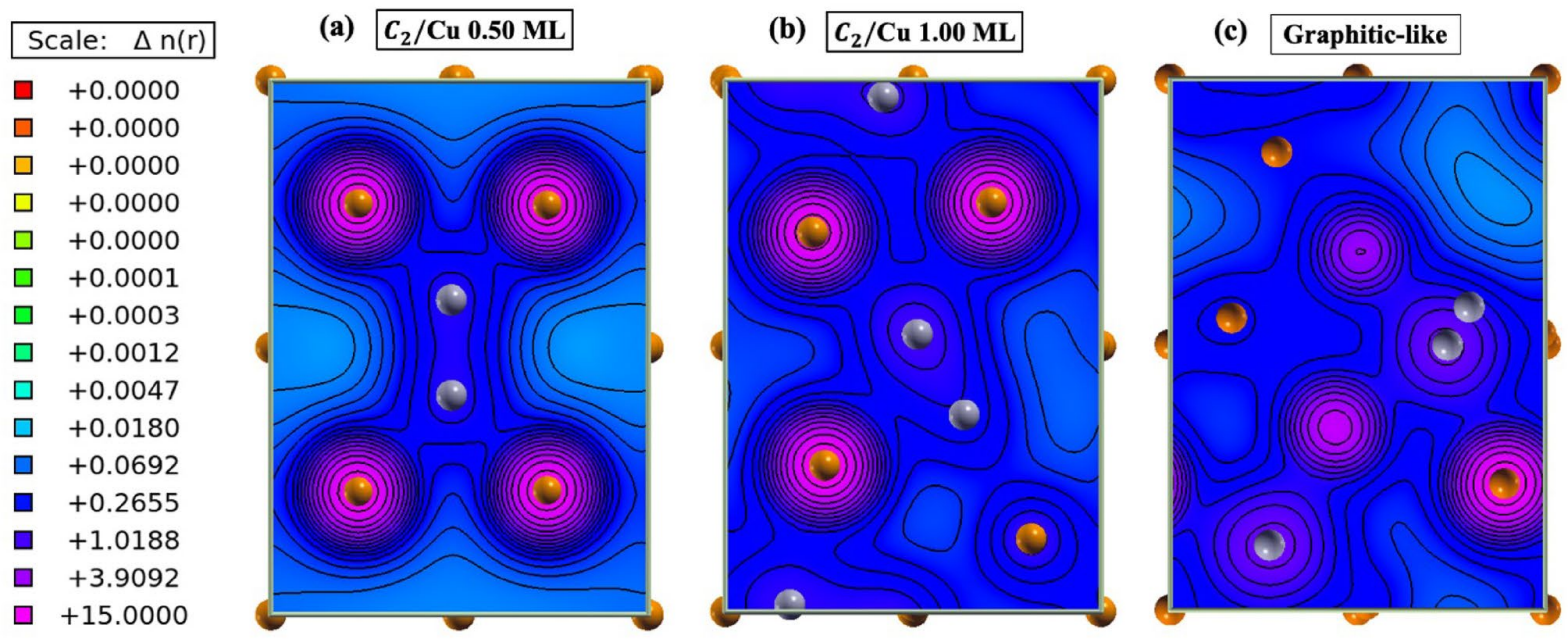
Calculated charge density [n(r) (e/Å$^3$)] contour plots for the outermost layer of C$_2$/Cu at (**a**) 0.50 and (**b**) 1.00 ML coverages and (**c**) the graphitic-like structure. Gold and gray spheres represent the Cu and C atoms, respectively.

Thus, the calculated SEY shows the following trend: graphitic-like/Cu < C$_2$/Cu (1.00 ML) < C/Cu (0.25 ML) < C$_2$/Cu (0.50 ML). To understand the physical reason behind the observed SEY trend (Fig. 4c,d), the surface morphology of the Cu systems was investigated by studying the potential energy surfaces and charge densities of the outermost Cu layer. In Figs. 5 and 6, the two-dimensional potential energy surfaces and contour charge densities for the 0.50 and 1.00 ML C$_2$/Cu and graphitic-like systems are shown. As it can be seen in Figs. 5 and 6, formation of the C$_2$ pair in the 0.50 ML system created a strong C–C bond and reduced the bonding between the C and Cu nearest neighbors (contrary to the C/Cu systems), resulting in a higher SEY. By increasing the C–C coverage, the C$_2$ (Fig. 6b) and graphitic-like (Fig. 6c) adsorbates arrange themselves such that their bonding with surrounding Cu atoms is maximized resulting in surface deformation (Fig. 3). With this rearrangement, the bonding between the C and Cu atoms gets stronger in the 1.00 (Fig. 6b) ML C$_2$ and graphitic-like (Fig. 6c) structures with respect to the 0.50 (Fig. 6a) ML C$_2$/Cu system. One can expect that, as a result of the increasing C–C and Cu–C bonding, the surface potential energy of the systems will be significantly modified with respect to the clean Cu surface. The probability of escaping electrons from the low potential energy regions is less than regions of high potential energy. Therefore, systems with the largest combined regions of the low potential energy (blue color) should have the lower secondary emission. Comparing the low potential energy regions of the Cu systems (Fig. 5), one can see the following trend for the potential regions: graphitic-like < C$_2$/Cu (1.00 ML) < C/Cu (0.25 ML) < C$_2$/Cu (0.50 ML), which is consistent with the calculated and observed^9^ SEY trends previously discussed. It should be noted that the graphitic-like and 1.00 ML C$_2$/Cu systems have comparable amounts of low potential energy regions; however, the larger work function of the graphitic system (at 7.63 eV as listed in Table 1) results in a lower overall SEY.

The theoretical result of substantial SEY changes upon formation of graphitic carbon on the surface agrees with experimental results reported by Watts et al.^32^ where the surface of copper samples was modified using a high-power neodymium doped yttrium aluminum garnet laser, and where the degree of surface modification depended on the duration and intensity of the laser exposure. Four different levels of modification were tested, in addition to the unmodified sample. Minor modification resulted in the biggest effect, significantly reducing the SEY. It is very likely that high duration or intensities of laser exposure might have led to ablation, while surface graphitization resulted at the lower dose. These observed outcomes would then be consistent with our simulation predictions.

## Summary

Using a combination of first-principles, MC, and MD simulations, the effect of an atomic C, C$_2$ pair, and graphitic-like layer on the SEY of the Cu (110) surface was studied. For all systems, the input parameters for the SEY (kinetic MC simulation), including the work functions, densities of states, and dielectric functions, were calculated using first-principles methods. The calculated SEY revealed that the amount of C–C bonding present at the surface can greatly effect the number of secondary electrons. For increasing atomic C coverage, the SEY increased while the opposite affect was observed for the C$_2$ pair system. To confirm the effects of the electron irradiation on the C/Cu system, a MD simulation with the COMB3 interatomic potential scheme was performed and revealed the formation C$_2$ pairs from atomic C layers. Additionally, an energetically stable graphitic-like structure (six C layers) was studied. In agreement with experimental observation, it was shown that the graphitic-like structure, when compared to the other systems, had the largest SEY reduction with respect to the Cu (110) surface. This theoretical result of substantial SEY changes upon formation of graphitic carbon on the surface agrees with experimental results reported by Watts et al.^32^. Analysis of the two-dimensional potential energy surfaces and charge density contour plots revealed that the surface morphology of the Cu surface is directly responsible for the changing SEY.

## Methods

### First-principles calculations

All first-principles calculations were performed using the density functional theory suite VASP (Vienna Ab initio Package). The VASP code is a pseudopotential code that uses the projector augmented-wave (PAW) technique^33–36^. For all calculations, the Perdew, Burke, Ernzerhof parameterized generalized gradient approximation (GGA) exchange-correlation functional^37^ was used with a 600 eV kinetic energy cutoff. A k-point sampling of $5\times 5\times 1$ Monkhorst Pack mesh^38^ was employed for the a 40-atom 10-layer surface slab of Cu (110). The calculated bulk lattice constant was obtained using the Murnaghan equation of state^39^, determined to be 3.63 Å (3.61 Å^40^), and was used to relax all systems. For all the various systems, the top four layers were allowed to relax with the bottom six being held fixed. Additionally, the break condition for the self-consistent loop was set to $10^{-5}$ eV and the Hellman-Feynman force was less than 0.01 eV/Å for all surfaces.

### Monte Carlo simulation

The kinetic Monte Carlo (MC) scheme used here consisted of 100,000 electrons. This choice of primary electrons yields an accuracy of $10^{-5}$ in electron yield of MC simulations. The MC simulation required a number of input parameters including the work function, total density of states, and dielectric function (real- and imaginary-parts of the frequency dependent permittivity). All input parameters were calculated using first-principles methods. This MC implementation uses the usual drift-and-scatter sequence with some incident electrons being backscattered elastically. Once an electron has entered into the material, it will travel a path length $L_i$ before interacting with another electron in the material. $L_i$ is obtained from the product of the energy dependent inelastic mean free path $\lambda _e(E)$ (Eq. 4) and a random number $r_i$ [i.e., $L_i=- \lambda _e(E)ln(r_i)$]. Combining $L_i$ with the stopping power (Eq. 5) the energy the electron loses ($\Delta E$) is obtained [$\Delta E=L_i\times (dE/dR)$]. The scattering process is then repeated with the new electron energy, with some electrons heading out of the material.

The MC simulation assumes collisions with shell electrons or free electrons, both of which are referred to as secondary electrons. As stated previously, when a collision occurs, the traveling electron loses energy $\Delta E$ that is given to the secondary electron. If this energy is greater than the binding energy ($E_b$) of a shell electron ($\Delta E-E_b > 0$), a collision with the shell electron is considered and is knocked free with total kinetic energy equal to $\Delta E-E_b$. If $\Delta E-E_b < 0$, then this interaction is treated as a collision with a free electron which now has total kinetic energy equal to $\Delta E$. Once any secondary electron is knocked free, it follows the drift-and-scatter sequence that may produce additional electrons or leave the material entirely. Additional, information on the implementation of the this MC scheme has been reported elsewhere^41^.

### Molecular dynamics simulation

The MD code LAMMPS (Large-scale Atomic/Molecular Massively Parallel Simulator)^42^ was used to simulate the effects of electron irradiation^43^ on the Cu (110) surface. The interactions between C–Cu, C–C, Cu–Cu, and C$_2$-Cu atoms were modelled using the third generation of the Charge-Optimized Many-Body (COMB3) interatomic potential^44^. The C/Cu and C$_2$/Cu structures created for DFT calculations were used as the basis to generate the supercells, with periodicity $20\times 14\times 1$, for the MD simulations. The number of surface C atoms for the C/Cu and C$_2$/Cu systems were taken to be 280 and 560, respectively. The geometry of the supercell was optimized at 0 K and was later relaxed at 300 K for 1 ns (time-step of 1 fs) with Nose-Hoover thermostat (NPT)^45^ under pressure 1 atm. For modelling the irradiation, the energy of the irradiated atoms was increased at a rate of $1.72\times 10^{-3}$ eV/ps with the use of the Berendsen thermostat^46^ until the entire adsorbates are dissociated from the surface. The remaining Cu atoms were kept at 300 K during the entirety of the MD simulations.

## Data Availability

The data that support the findings of this study are available from the corresponding author upon reasonable request.
