# Author Correction: Importance of surface morphology on secondary electron emission: a case study of Cu covered with carbon, carbon pairs, or graphitic-like layers

**DOI:** 10.1038/s41598-023-37839-x

**Published:** 2023-07-03

**Authors:** L. Diaz, A. Karkash, S. Alsharari, R. P. Joshi, E. Schamiloglu, M. Sanati

**Affiliations:** 1 Department of Physics and Astronomy, Texas Tech University, Lubbock, TX 79409 USA; 2 Department of Electrical and Computer Engineering, Texas Tech University, Lubbock, TX 79409 USA; 3 Department of Electrical and Computer Engineering, University of New Mexico, Albuquerque, NM 87131 USA

Correction to: *Scientific Reports* 10.1038/s41598-023-34721-8, published online 22 May 2023

The original version of this Article contained an error in the Acknowledgements section. The first grant number was incorrectly written.

“The work at Texas Tech University was supported in part by grants from the Air Force Office of Scientific Research (No. FA9550-19-1-0056) and the Office of Naval Research (No. N00014-22-1-2483). The work at the University of New Mexico was supported by AFOSR MURI Grant FA9550-21-1-0367 via a subaward from Michigan State University and ONR Grant N00014-23-1-2072. We also acknowledge the generous amounts of computer time provided by Texas Tech University High Performance Computer Center.”

now reads:

“The work at Texas Tech University was supported in part by grants from the Air Force Office of Scientific Research (No. FA9550-22-1-0499) and the Office of Naval Research (No. N00014-22-1-2483). The work at the University of New Mexico was supported by AFOSR MURI Grant FA9550-21-1-0367 via a subaward from Michigan State University and ONR Grant N00014-23-1-2072. We also acknowledge the generous amounts of computer time provided by Texas Tech University High Performance Computer Center."

The original Article has been corrected.

